# High resolution and sensitivity gamma camera with active septa. A first Monte Carlo study

**DOI:** 10.1038/s41598-019-54934-0

**Published:** 2019-12-05

**Authors:** Victor Ilisie, Laura Moliner, Sandra Oliver, Filomeno Sánchez, Antonio J. González, Michael Seimetz, Maria J. Rodríguez-Álvarez, Jose Maria Benlloch

**Affiliations:** Instituto de Instrumentación para Imagen Molecular, Camino de Vera s/n 46022, Universidad Politécnica de Valencia - CSIC, 46022 Valencia, Spain

**Keywords:** Biotechnology, Cancer, Diseases, Health care, Medical research, Physics

## Abstract

Gamma cameras are of great interest due to their high potential in the field of Nuclear Medicine Imaging. They allow for an early diagnosis of reduced size tumors, and also for a wide variety of preclinical studies with the aim of designing more effective treatments against cancer. In this work we propose a significantly improved multi-pinhole collimator gamma camera and perform a first Monte Carlo analysis of its characteristics. Maintaining the configuration of a multi-pinhole collimator with a high degree of overlapping (thus with a high sensitivity), we add a new element, an *active septa*, that besides acting as a collimator, is able to measure the impact coordinates of the incident photon. This way one is able to unambiguously identify through which pinhole any gamma ray passes before being detected. The result is a high sensitivity and resolution multi-pinhole gamma camera with an arbitrarily large field of view. As a consequence, the final reconstructed image does not suffer from the undesired artifacts or truncation associated to the multiplexing phenomenon. In this study we focus on the development of a system able to visualize in 3D tumors, nodes and metastasis in real time in the operating room with very low dose. We also briefly analyse and propose a novel design for a Single Photon Emission Computed Tomography system.

## Introduction

Gamma cameras have been mainly used in medicine for diagnosis purposes, however, in some cases they are also used in the operating room. Therefore, gamma cameras are interesting, because surgeons are used to perform gamma probes for localizing and extracting lymphoid nodes during surgery procedures. A main drawback of this technique is that the surgeon has to wait more than a minute for an image to be formed, and this is time consuming as the operation has to be repeated many times, before finding the region of interest. This translates into *dead-time* that is spent in the operating under gamma-ray radiation. Our current project, aims at solving this particular problem.

**Figure 1:**
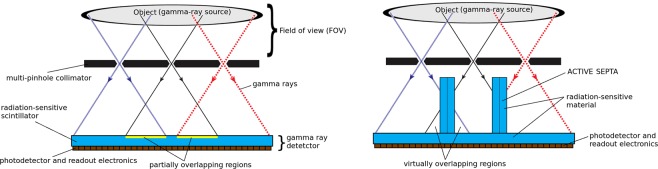
Left: multi-pinhole gamma camera with overlapping regions in the detector. Right: schematic representation of a multi-pinhole collimator gamma camera with active septa.

A common problem of gamma cameras is their reduced sensitivity. Only the gamma rays that are emitted parallel to the collimators (for a gamma camera with parallel collimators) or within a certain angular region (for a gamma camera with pinhole collimators) are the ones that reach the detector. In order to increase the sensitivity of a pinhole collimator gamma camera, one necessarily has to increase the number of pinholes and/or the angular aperture. When increasing the number of pinholes one is faced with the overlapping/multiplexing problem, as shown schematically in Fig. 1(left). Within the overlapping region one cannot identify unambiguously through which hole the incident gamma ray has previously passed before being detected. The image reconstruction technique in emission and transmission tomography is based on tracing lines of response (LORs) through the field of view (FOV). In the case of pinhole gamma cameras the LORs are built by joining the detected impact point of the gamma ray with the corresponding pinhole (through which it has previously passed). Not being able to identify the correct pinhole in the overlapping region and making all possible combinations, translates into considering wrong LORs in the image reconstruction process. This introduces noise into the final reconstructed image and possible artifacts^1–3^.

There are also issues associated to detectors with collimators that produce no overlapping. Some of them are image truncation^4^ and a more reduced FOV that contains blind regions. A great number of studies have been performed to improve gamma cameras and Single Photon Emission Computed Tomography (SPECT) performance and remove overlapping effects^1–4^. However no generic solution has been found as the problem highly depends on the complexity of the object of study (small animals, organs, etc.), FOV, desired resolution, and many other design parameters. One practical solution that offers a high sensitivity and resolution SPECT/gamma camera has been proposed in ref. ^5^, where the collimator presents a great number of holes and has cylindrical shape. However it has a serious drawback, a very reduced FOV. Therefore this type of devices can only be used for preclinical studies with small animals. Also due to the reduced FOV, in order to study a wide region of the animal’s body, the animal has to be moved during the scan.

Alternatively, coded aperture masks have been proposed to increase sensitivity of gamma cameras, while maintaining spatial resolution capabilities of the system^6–9^. In these designs, the collimator is replaced by a mask with a great number of holes, following a special arrangement that allows some decoding procedure to recover an image. Such coded masks have been designed and used for several decades, mainly for astrophysical applications and monitoring stationary nuclear materials in nuclear power plants and nuclear fuel reservoirs. For this last application active coded masks providing electronic collimation based on Compton kinematics (the so-called Compton camera) and mechanical collimation are combined to increase sensitivity^9–11^. However, for Nuclear Medicine applications, where the object under study should be placed as close as possible to the detector plane, the near-field artifact produced by such reduced distance imposes a big challenge to the images obtained with coded aperture mask systems, making it necessary to implement mask-antimask techniques to partially overcome the problem^12^.

The main objective of this Monte Carlo analysis, is the study of the development of a breakthrough Molecular Imaging System able to visualize in 3D tumors, nodes and metastasis in Real Time in the operating room and with very low dose. Based on the presented theoretical results, a real gamma camera device will be finally developed in the forthcoming years. It consists in an ultra high sensitivity and resolution multi-pinhole gamma camera device that eliminates the multiplexing/overlapping problem (associated to multi-pinhole collimators) without producing image truncation or any artifact. Maintaining the configuration of a multi-pinhole collimator with a high degree of overlapping (providing thus a very high sensitivity) we add a new element, which we called *active septa*^13^, which is a radiation sensitive detector that acts both as a detector and a collimator, as shown in Fig. 1(right). It prevents the gamma rays to reach the overlapping region and at the same time it measures the impact coordinates of the incident photon. This way all the needed information is retrieved and we are able to unambiguously identify through which pinhole any gamma ray passes before being detected.

## Monte Carlo Analysis

The active septa can be formed by scintillating crystals provided with their own photodetectors and corresponding electronic readout. However, in a simplified version, which is the one that we are going to analyse here, they do not have their own photodetectors. The crystals are coupled directly to the bottom crystal and they are covered in (optical) reflective material such as ESR, in order to guide the optical rays towards the bottom detector, where the readout is performed. Further on, we are also going to analyse the proposed method that can be used in order to recover the impact coordinates for such configuration.

**Figure 2:**
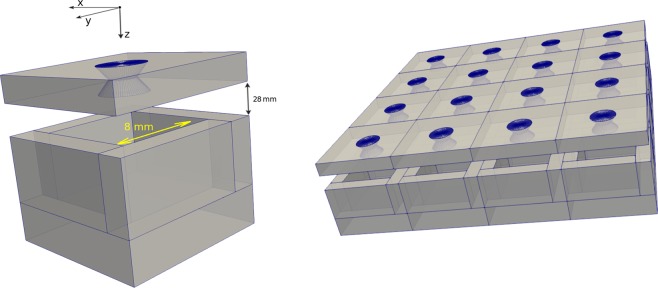
Left: basic module of a multi-pinhole gamma camera (scintillators and pinhole collimator) together with the global coordinate system, where the coordinate origin is placed at the center of the pinhole, at a distance of 45.2 mm (in z) from the collimator. Right: final gamma camera system composted of a 4 × 4 array array of basic modules. The corresponding detector geometry was generated with GATE^24,25^ and the figure was plotted with ParaView (https://www.paraview.org/).

For this simulation study, the whole gamma camera system will be composed of 16 basic modules (see Fig. 2). Each module is composed of four active septa *walls* of $2\times 10\times 5\,{{\rm{mm}}}^{3}$ coupled to a $12\times 12\times 3\,{{\rm{mm}}}^{3}$ bottom detector. These sizes approximately correspond to the basic module that will be finally developed. We have considered GaGG scintillator as the detector material. The pinhole collimator is a Tungsten block of 2 mm thickness, with a 2 mm (inner) diameter and a 40° opening.

**Figure 3:**
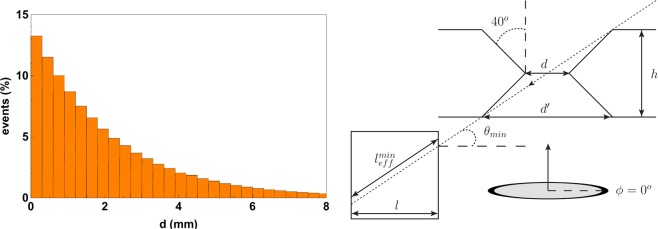
Left: Percentage of events (first interactions) for a gamma ray source of normal incidence on a large GaGG block as a function of the depth of interaction *d* (mm). Right: details of the configuration of a basic module of the gamma camera. In our case the thickness of the pinhole collimator is $h=2\,{\rm{mm}}$, the inner diameter is $d=2\,{\rm{mm}}$, the outer diameter $d^{\prime} \simeq 3.68\,{\rm{mm}}$. The thickness of the active septa is $l=2\,{\rm{mm}}$. An incident gamma ray for $\phi =0^\circ$ and $\theta ={\theta }_{min}$ is also shown.

In Fig. 3 (left) we show a histogram with the percentage of events that suffer their first interaction at depth *d* (mm) for a 140 keV gamma ray source, for a large and thick GaGG scintillator block and central normal incidence. We can observe that roughly 80% of the first interactions occur within the first 3 mm. From a simple statistical analysis we can also conclude that the photoelectric effect dominates at these energy levels and it sums about 85% of the total number of interactions. This translates into a very high efficiency of the detector. It means that the stopping power of the bottom detector should be around 80% or greater (depending on the incidence angle). Similar conclusions can be reached for the active septa. As the incidence angle is at least ${\theta }_{min}=28.5^\circ$ (see Fig. 3 (right) for details), we can define a minimum effective thickness of the wall as ${l}_{eff}^{min}=l/\cos ({\theta }_{min})\simeq 2.3\,{\rm{mm}}$, which translates into 67% stopping power. However, this effective length is greater for greater *θ* and also for $\phi \ne 0^\circ ,90^\circ ,180^\circ ,270^\circ$. Therefore we expect a small amount of noise from the gamma rays that go through the active septa. This noise can be removed by additionally adding a thin plate of Tungsten in between the active septa.

**Figure 4:**
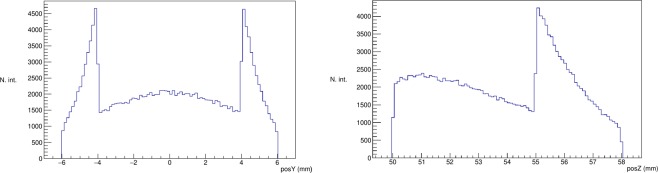
Histograms of the number of interactions as a function of the *z* (left) and *y* (right) impact coordinates.

We can determine the gained sensitivity (with no multiplexing) obtained by introducing the active septa from the Monte Carlo simulation. Placing a large spherical source of radius 10 cm with its center placed at 10.5 cm from the pinhole collimator we plot the obtained interaction histograms for the *y* and *z* axes in Fig. 4 (a plot similar to the one corresponding to the *y* axis is obtained for the *x* axis). For the *z* axis, the impact coordinates in between 55 and 58 (mm) correspond to the bottom detector and the ones in between 50 and 55 (mm) correspond to the sum of the impacts in all four active septa. Similarly for the *y* axis, the impacts with coordinates in between −4 and 4 (mm) correspond to the interactions with the bottom detector and the ones in between −6 and −4 and, 4 and 6 (mm) correspond to a mixture of the interactions in all four active septa and the bottom detector. Out of the total number of interactions the ones that occur in any of the five detectors, approximately 60% occur in the active septa, which is the gain estimation introduced by the additional detectors. However, the overall gain factor can actually be higher when compared to gamma cameras with only one bottom detector (available in the market). As the current device is modular, there is no physical limit on the number of modules that can be attached to the gamma camera. In the case of traditional gamma cameras with an unique detector, there is a technological limit corresponding to the growth process of the scintillator crystals, and a second limiting factor corresponding to the elevated price of large crystal blocks.

### Spatial resolution

In order to determine the spatial resolution of both the bottom detector and the active septa, Monte Carlo optical simulations have been performed. For this part, only one gamma camera module is used. An isotropic gamma ray point source is placed at the center of the pinhole, and the positions of the gamma ray interactions are used to generate optical photons. The detection of the optical photons is performed by the photodetector placed at the bottom. A high performance detector setup can be experimentally implemented with current Silicon photomultiplier (SiPM) technology coupled to monolithic scintillator blocks^14^. Thus, in order to mimic this behaviour we will simulate a 3 × 3 mm^2^ detector readout with 50% quantum efficiency.

Several types of optical finishing surfaces have been simulated. The best results were obtained with specular reflector (such as ESR) for the two large sides of the active septa with its two lateral small sides black-painted, and with a retro-reflecting layer on the top. As for the bottom detector, the best configuration was obtained with all four lateral sides black-painted and retro-reflecting layer on its top, except the shared area between the active septa and the bottom detector which is coupled with optical grease.

In order to reconstruct the impact coordinates from the detected optical photons, several methods have been tested. Both the center of mass (CM) and its Raise to Power (RTP) extension^15^, fail in retrieving useful information from the active septa. The resulting reconstructed impact coordinates are always placed at the center or close to the center of the detector. Another method for the coordinate estimation that can be employed in our case (for relatively thin detectors, coupled to a larger detector) is by assigning the coordinates of the pixel of maximum charge (energy). This method also gives rather poor results. In sight of the previous outcomes we have developed a novel method that consists in calculating the center of mass of the first *N* maxima, with *N* a positive integer that needs to be empirically determined. This is

1 $$\bar{x}=\frac{{\sum }_{i=1}^{N}\,{x}_{i}\,{Q}_{i}^{max}}{{\sum }_{i=1}^{N}\,{Q}_{i}^{max}},\,\bar{y}=\frac{{\sum }_{i=1}^{N}\,{y}_{i}\,{Q}_{i}^{max}}{{\sum }_{i=1}^{N}\,{Q}_{i}^{max}},$$

where ${Q}_{i}^{max}$ with $i=1,\ldots ,N$ are the first *N* maxima and where $\bar{x}$ and $\bar{y}$ are the estimated coordinates. In order to better understand the previous definition of *N* maxima consider the set composed by the 4 × 4 elements, *Q*_*i*_, with $i=1,\ldots 16$, that correspond to the charge deposited in each pixel of the photodetector. If we order this set descendently in an array, the first *N* maxima are the first *N* elements of this list.

**Figure 5:**
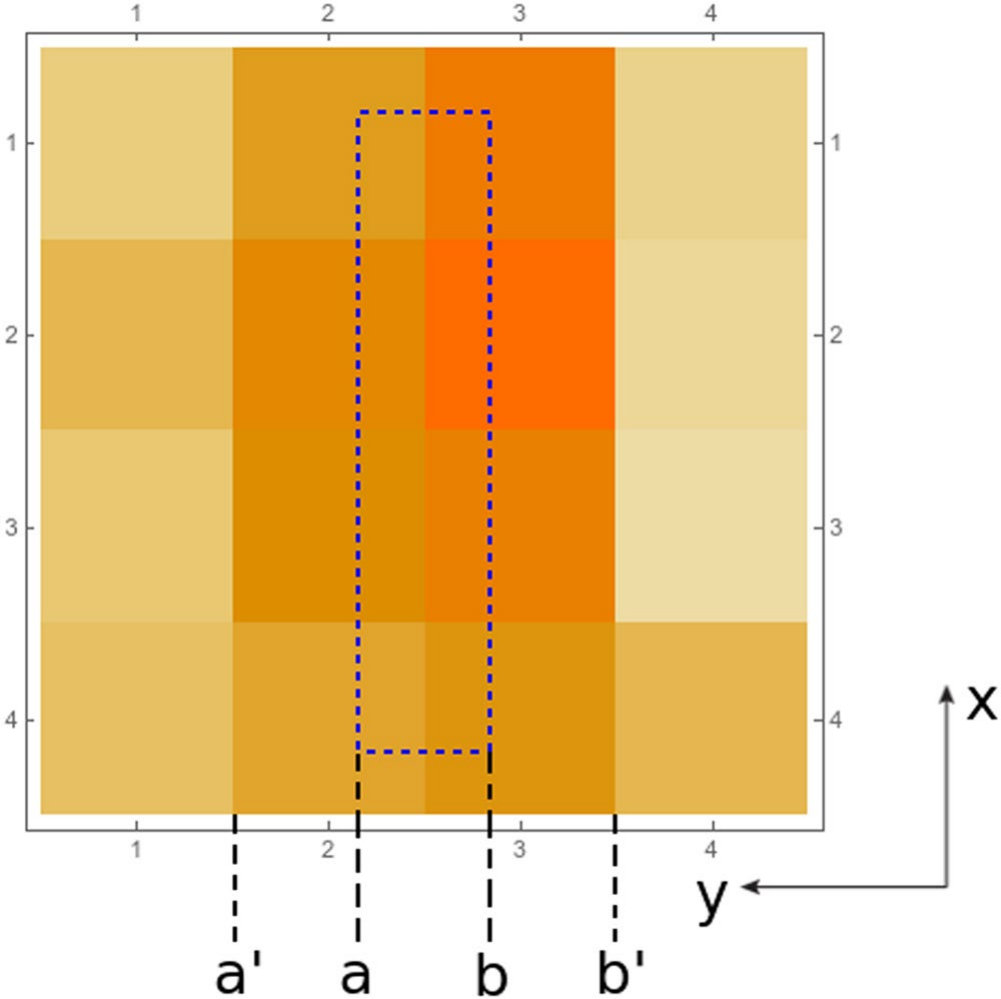
Top view of the placement of the active septa with respect to the pixelated photodetector.

A top view of how the detector is placed with respect to the photodetector pixels is shown in Fig. 5. By placing the active septa as in the figure (with two rows of pixels underneath it, instead of one) one can extract useful information also along the *y* axis. Note that by using the previous expression the reconstructed *y* coordinate can fall outside the upper detector i.e., in the interval $[a^{\prime} ,b^{\prime} ]$. We thus have to re-normalize the interval $[a^{\prime} ,b^{\prime} ]$ to $[a,b]$ in order to always obtain the coordinates inside the (active septa) detector.

**Figure 6:**
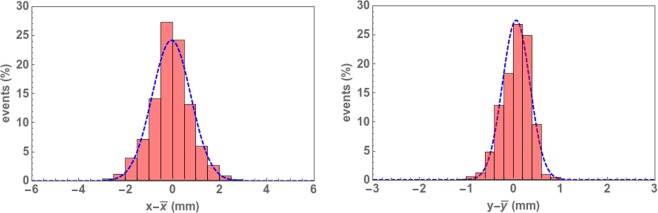
Histograms distributions of $x-\bar{x}$ and $y-\bar{y}$ where *x* and *y* are the *real* impact coordinates and $\bar{x}$ and $\bar{y}$ are the estimated coordinates. The corresponding Gaussian fit is also shown in each case by the dotted curve.

With these considerations, we obtain the results shown in Fig. 6 for the *x* and *y* spatial resolution. The histograms correspond to the number of events (in percentage) as a function of the difference between the real interaction position (obtained from the simulations) and the corresponding estimated interaction coordinates, by using the expressions from (1). The blue dashed line corresponds to a Gaussian fit. The best results were obtained for $N=4$ i.e, by employing the first four maxima. The fitted distributions correspond to ${\sigma }_{x}\simeq 0.8\,{\rm{mm}}$ (1.9 mm FWHM) for the *x* coordinate and ${\sigma }_{y}\simeq 0.3\,{\rm{mm}}$ (0.7 mm FWHM) for the *y* coordinate.

Let us now move to the depth of interaction (DOI) estimation. In this work we shall use the ${\mathscr{N}}/ {\mathcal I}$ estimator^16^. It corresponds to ${Q}_{tot}/{Q}_{1}^{max}$ (using our current notation), where the total charge *Q*_*tot*_ is simply given by

2 $${\mathscr{N}}={Q}_{tot}=\mathop{\sum }\limits_{i=1}^{16}\,{Q}_{i},$$

where ${\mathcal I} ={Q}_{1}^{max}$ corresponds to the first maximum, or simply the maximum value of the charge deposited in a pixel for an event. For the same previous simulation with an external isotropic gamma ray source we calculate the difference between the estimated DOI and the real one (obtained from the simulation). The obtained distribution corresponds to a Gaussian-like distribution with $\sigma \simeq 1.48\,{\rm{mm}}$ (3.5 mm FWHM). As we will see in the next section, for our purposes of reconstructing sources of diameter of about 1 cm the obtained resolution seems to be just enough.

For the bottom detector the reconstruction of the $(x,y)$ coordinates is performed the standard way by using the CM (RTP2), and no DOI information is estimated. The estimated resolution in this case is approximately 1.2 mm (FWHM).

In order to determine whether the interaction took place in the active septa or in the bottom detector for the events that fall in the overlapping $(x,y)$ region we use the DOI estimator together with the Anger logic as follows. If the estimated DOI falls in the active septa region and the reconstructed CM for the $(x,y)$ coordinates is close to the center of the active septa, then we consider that the event took place in the active septa and we use (1) in order to obtain the $(x,y)$ coordinates. The calculated efficiency of this method is more than 90%.

### Image reconstruction method

Before presenting the results, which are reconstructed images of simulated Derenzo phantoms, we need to introduce the image reconstruction method. Here we use the list-mode ordered-subsets expectation-maximization (LM-OSEM) algorithm^17–20^, based on ordered sets of events rather than indexed LORs. As mentioned previously, in our case a LOR corresponds to the straight line that passes through the point given by the impact coordinates of the gamma ray (in the detector) and the center of the corresponding pinhole collimator. For the reconstruction process we divide the whole set of non-indexed LORs into *L* subsets and call *S*_*l*_ $(l=1,\ldots ,L)$ the *l*th subset. The image estimate ${\lambda }_{j}^{m,l}$ (at voxel j, *m*th iteration and *l*th subset) will be then given by^20^

3 $${\lambda }_{j}^{m,l}=\frac{{\lambda }_{j}^{m,l-1}}{{\sum }_{i\in I}\,{p}_{ij}}\,\sum _{k\in {S}_{l}}\,{p}_{{i}_{k}j}\frac{1}{{\sum }_{b\in J}\,{p}_{{i}_{k}b}\,{\lambda }_{b}^{m,l-1}},$$

where

4 $$I={\cup }_{l\in L}\,{S}_{l},$$

is the set of all LORs, *i*_*k*_ is the LOR corresponding to the *k*th event, *J* is the set of voxels traversed by the LOR *i*_*k*_, and finally *p*_*ij*_ is the probability of an emission from voxel *j* being detected along the LOR *i*. In order to obtain *p*_*ij*_ we use Jacobs’ implementation of Siddon’s algorithm^21,22^. Finally, one needs an estimation of the elements ${\sum }_{i\in I}\,{p}_{ij}$. For an indexed-LOR approach and virtual pixels, the procedure is straightforward, as there is finite number of possible LORs. Here we shall use a slightly different approach based on Monte Carlo simulations in order to avoid the use of virtual pixelization of the detectors. A uniform virtual photon source with large activity and the size of the whole FOV is placed within the FOV. After the acquisition, a very large set of LORs is obtained, of the order of 10^8^. The elements ${\sum }_{i\in I}\,{p}_{ij}$ are then estimated by using (Jacobs’ implementation of) Siddon’s algorithm.

This approach can be regarded as a practical solution for avoiding LOR-indexing and virtual pixeling of the detector. As it will be presented in the following, high-quality images are obtained with this algorithm implementation. After each iteration or (sub-iteration) a Gaussian smoothing is applied^23^.

## Results and Discussion

Our aim is to explore the resolution and possibilities that the proposed device can offer for obtaining real-time images of ganglia, nodes or tumors of diameter of about 1 cm. For this purpose we have simulated a sphere (as background radiation) of 4 cm radius and centred in the center of the FOV, with a total activity $A=3.7\,{\rm{MBq}}$, which translates into a specific volume activity

5 $${A}_{V}=A/Vol\simeq 0.373\,\mu {\rm{Ci}}/c{m}^{3}.$$

**Figure 7:**
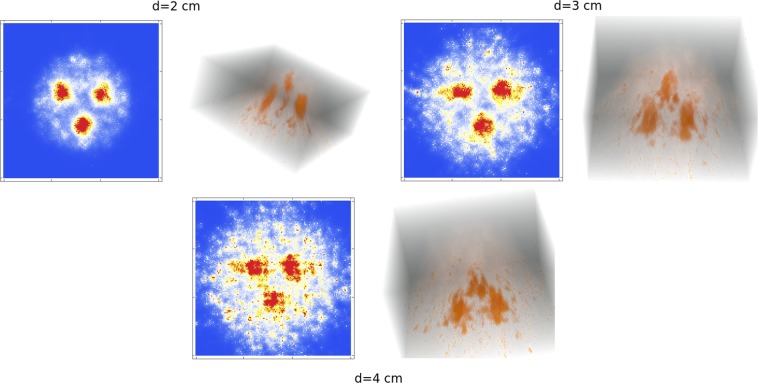
Reconstructed images, in 2D and 3D, of a Derenzo phantom with 3 spherical sources of 1 cm diameter with a spherical background of 8 cm diameter, for several values of the distance *d* (between the Derenzo phantom and the collimators) and a 10 s acquisition time. The reconstructed images were obtained with a C++ routine implemented by V.I. and the images were plotted with Mathematica (https://www.wolfram.com/mathematica).

Inside this sphere we placed a Derenzo phantom composed of three smaller spheres, of 1 cm of diameter (and 2 cm distance in between their centres) and specific volume activity ratio 2:1. The three spheres were placed forming a plane parallel to the pinhole collimator plate. The distance *d* of the plane formed by the three spheres and the pinhole collimators was then varied. The results for a 10 s acquisition are shown in Fig. 7 for $d=2\,{\rm{cm}}$ (top-left) $d=3\,{\rm{cm}}$ (top-right) and $d=4\,{\rm{cm}}$ (bottom) where, together with the 2D slices we also present full 3D images. One can observe that the resolution is obviously more degraded for larger values of *d*, however one can still appreciate the three spheres in all cases. The *pixelization* of the last image can be appreciated more significantly and it is due to the lack of DOI information for the bottom detector. Thus, as for tumour location searching, lesser quality images are sufficient, 10 s or less would be more than enough in order to localize the region of interest (ROI).

**Figure 8:**
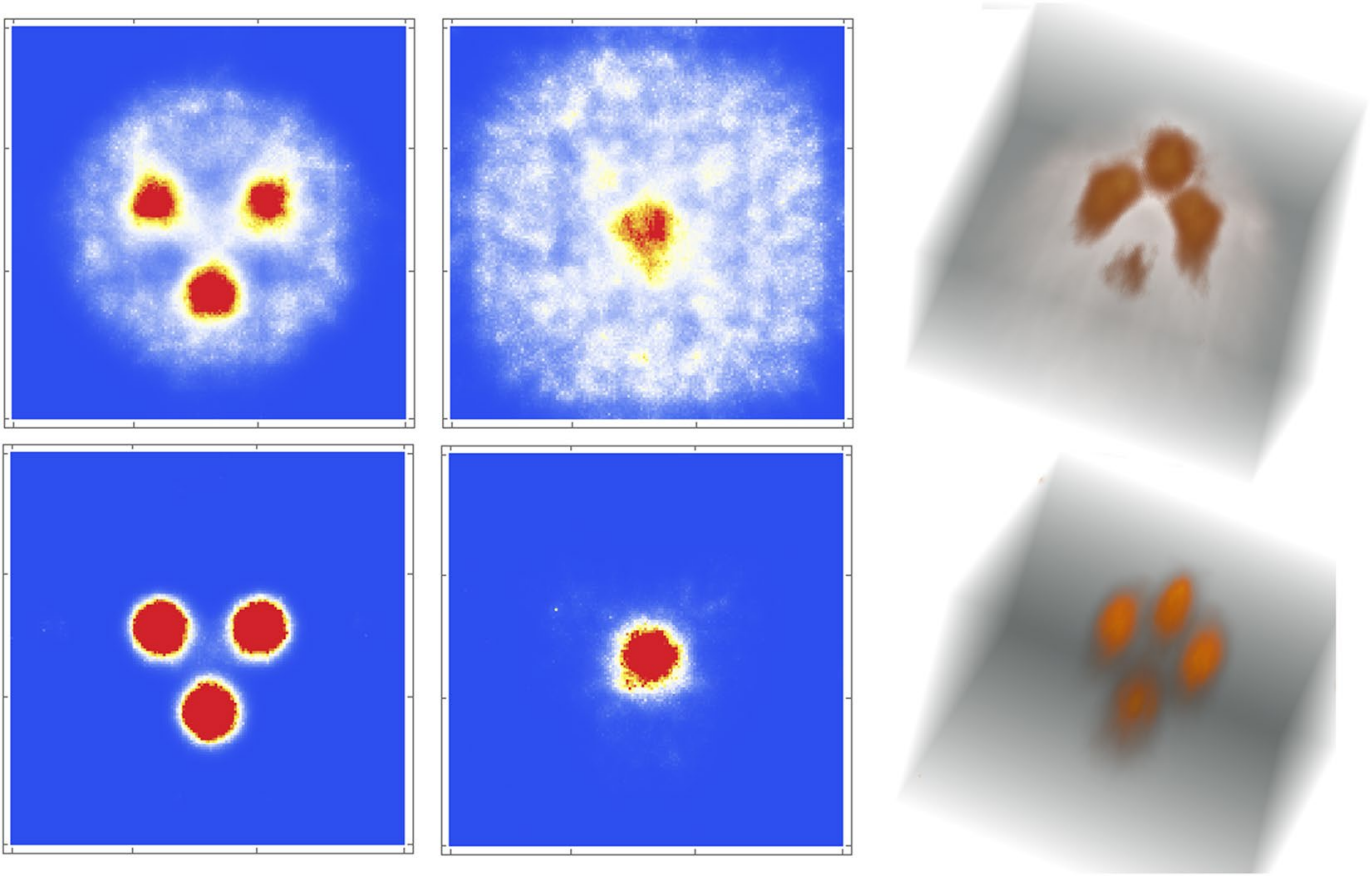
Reconstructed images, in 2D and 3D, of a the tetrahedral Derenzo phantom with 4 spherical sources of 1 cm diameter with a spherical background of 8 cm diameter (top) and with no background (bottom) and 60 s acquisition time. The reconstructed images were obtained with a C++ routine implemented by V.I. and the images were plotted with Mathematica (https://www.wolfram.com/mathematica).

Once found the ROI, slightly increasing the acquisition would be more than enough to obtain interesting details of the tumors. In order to demonstrate this, we also show in Fig. 8 the image reconstruction of a 60 s acquisition of a 3D Derenzo phantom formed by a tetrahedron of spheres of 1 cm diameter and 2 cm distance in between their centres, with the same background as previously (top) and with no background (bottom). The distance of the plane (formed by the three spheres) that is parallel to the pinhole collimator is 2 cm.

With the previous analysis we have demonstrated that the novel proposed apparatus can indeed be a very useful tool for medical purposes in live monitoring. With slightly more increased statistics (about 1 min) the quality of the obtained image increases substantially thus providing more accurate information about the tumour position.

### Future SPECT proposal

**Figure 9:**
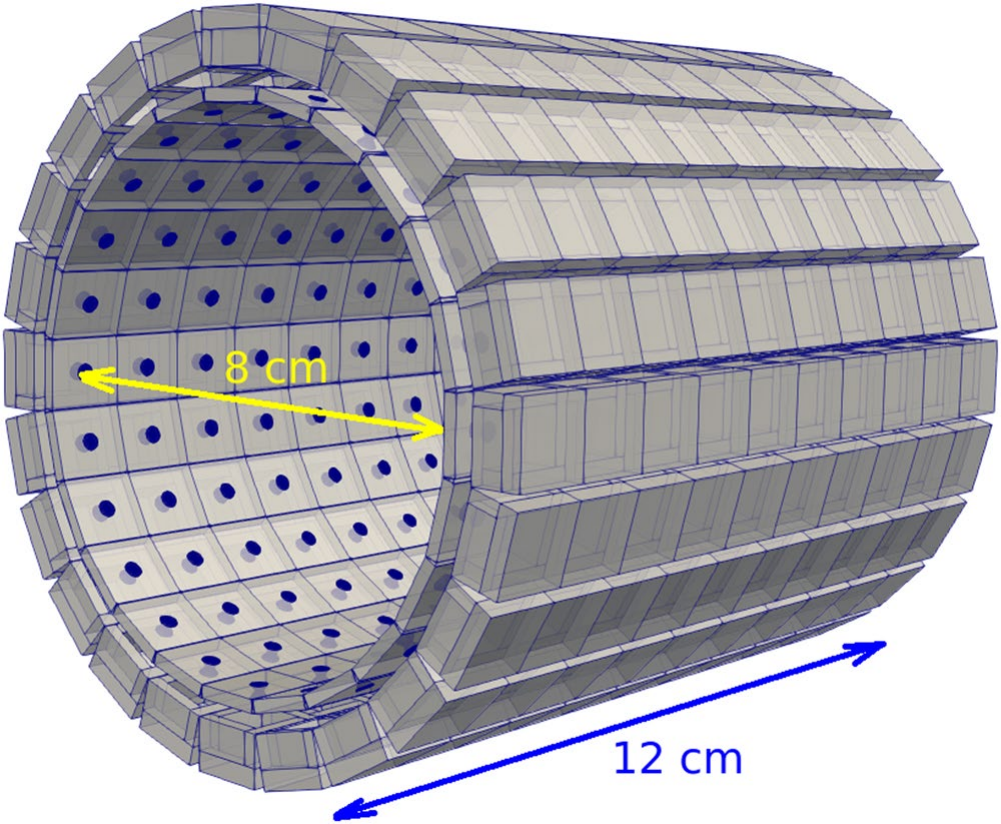
A 240 module proposed SPECT device for preclinical studies with small animals. The corresponding detector geometry was generated with GATE^24,25^ and the figure was plotted with ParaView (https://www.paraview.org/).

In order to fully benefit from the full potential of the current modular gamma camera, we propose a novel cylindrical SPECT apparatus formed by 240 modules, distributed as shown in Fig. 9. For this configuration it has approximately 8 cm of inner diameter and 12 cm of length. It can be used for preclinical studies with small animals. This geometry presents a great deal of benefits. One of these benefits is a highly increased sensitivity. For a regular SPECT device that rotates around the object of study/animal/patient, the angular coverage for each acquisition position is very limited. In our case, the angular coverage is 360°. Another great benefit is the need of a less complex rotation device. The pinholes are placed at 15° interval, therefore, one can cover all acquisition angles by mostly rotating the device in small steps up 15°.

**Figure 10:**
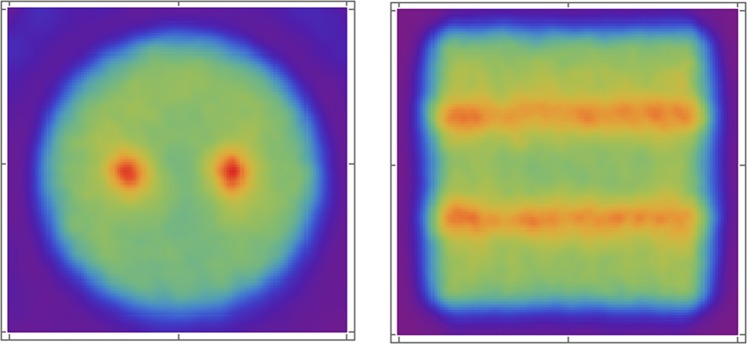
Image reconstruction of a cylindrical phantom with the previously proposed SPECT device for a 50 s acquisition time, 5 rotations (each 3°) and with no spatial blurring introduced. The reconstructed images were obtained with a C++ routine implemented by V.I. and the images were plotted with Mathematica (https://www.wolfram.com/mathematica).

As a final part of this study, to demonstrate the potential of this novel SPECT device, we reconstruct a phantom formed by a hot cylinder (placed along the *z* axis of the detector) of 5 cm diameter and 5 cm length, and two additional hot cylinders (with 10:1 specific activity ratio) of 2 mm diameter and same length. The total activity of the big cylinder is 3 MBq, and the acquisition time is rather short, 10 seconds for each position (the device was rotated in steps of 3°). The reconstructed image is shown in Fig. 10. No artifacts are appreciated. No spatial blurring was introduced. The only source of noise is due to the small percentage of gamma rays that pass through the active septa, interacting in the neighbour cell. The extra image blurring is mostly due to the low statistics of the acquisition. Further studies are of course needed in order to assess the full potential of the proposed SPECT device.

## Conclusions

In this work we have introduced a new concept in gamma ray imaging by presenting a high sensitivity and resolution multi-pinhole gamma camera free of the issues associated to the multiplexing phenomenon. We have thus proposed a practical solution to the multiplexing problem, the active septa, which is an additional detector that prevents the gamma rays from reaching the overlapping region and that brings a considerable increase in sensitivity.

We have presented a novel approach for impact coordinate estimation adapted to the basic cell geometry and characteristics of the proposed gamma camera. From the previous considerations we can conclude that the proposed apparatus presents an increased sensitivity, mainly due to its high angular coverage, sensitivity per cell and number of cells. Further studies have to be performed, however, as the sensitivity of the gamma increases with respect to current technology, a corresponding dose reduction will be possible. This could open the application of Molecular Imaging to special patients such as babies or children.

We have shown that the new camera can be provided with real time imaging as it is able to obtain a relatively high quality image in about 10 seconds or less. The current design of the camera makes it portable and easy to incorporate in the operating room. We have shown that 3D images are possible with a planar gamma camera without rotation. Due to this multiple-eyes technology, one should be able to get 3D details of the region of interest with this camera.
