# Author Correction: High resolution and sensitivity gamma camera with active septa. A first Monte Carlo study

**DOI:** 10.1038/s41598-020-68816-3

**Published:** 2020-07-14

**Authors:** Victor Ilisie, Laura Moliner, Sandra Oliver, Filomeno Sánchez, Antonio J. González, Michael Seimetz, Maria J. Rodríguez-Álvarez, Jose Maria Benlloch

**Affiliations:** Instituto de Instrumentación Para Imagen Molecular, Camino de Vera s/n 46022, Universitat Politècnica de València - CSIC, 46022 Valencia, Spain

Correction to: *Scientific Reports* 10.1038/s41598-019-54934-0, published online 05 December 2019

The original version of this Article contained an error in the Affiliation, which was incorrectly given as ‘Instituto de Instrumentación para Imagen Molecular, Camino de Vera s/n 46022, Universidad Politécnica de Valencia - CSIC, 46022, Valencia, Spain’. The correct affiliation is listed below:

Instituto de Instrumentación para Imagen Molecular, Camino de Vera s/n 46022, Universitat Politècnica de València - CSIC, 46022, Valencia, Spain.

This error has now been corrected in the HTML and PDF versions of the Article.

